# Cultivating the next generation of leaders: How postdocs, principal investigators and institutes can nurture and select for leadership competencies

**DOI:** 10.1038/s44319-024-00308-1

**Published:** 2024-11-12

**Authors:** James W Bryson, Ülkü Uzun, Victor O Oria, Jamie Y Auxillos, Iman Safari, Alexia M Lopresti, Agnieszka Krzyzanowska, Katrine Sonne-Hansen

**Affiliations:** 1 Biotech Research and Innovation Centre (BRIC), University of Copenhagen, Copenhagen, Denmark; 2 Section for Computational and RNA Biology, Department of Biology, University of Copenhagen, Copenhagen, Denmark

**Keywords:** Careers, Science Policy & Publishing

## Abstract

The transition from postdoc to PI is the most challenging career step. Peer groups, PIs and research institution can help postdocs to acquire the necessary skills and experience.

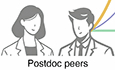

A recent survey of 3838 postdocs highlighted that, beyond financial factors, opportunities for career advancement was among the top five concerns of the respondents (https://www.nature.com/naturecareers/article/nature-2023-postdoc-survey). Notably, the biggest choke point for progression within academia is the leap from postdoc to principal investigator (PI). Research institutions have begun giving more attention to selecting candidates with the necessary skills to succeed within the role, but training postdocs to acquire these skills has only recently been prioritised. Given that institutions increasingly require that candidates demonstrate leadership skills and experience, the acquisition of said skills during postdoctoral training is becoming ever more essential for career progression. This is not only relevant for PI positions, but also for leadership positions outside academia.

“Research institutions have begun giving more attention to selecting candidates with the necessary skills to succeed within the role, but training postdocs to acquire these skills has only recently been prioritised.”

We represent postdoc fellows and PIs of the Marie Skłodowska-Curie COFUND Program ‘LEAD’ (https://lead.ku.dk) at the Biotech Research and Innovation Centre (BRIC), University of Copenhagen, as part of the European Union’s Horizon 2020 (H2020) Research and Innovation Programme. The programme started in 2021 and explicitly focuses on cultivating the next generation of “creative, collaborative, responsible and inclusive research leaders in either academia or industry”. The idea behind LEAD was encouraged by two observations. First, many postdocs at BRIC started reflecting on their next career step only during the last year of their position, which meant that acquiring new competencies became a battle against time. Second, whilst they remained interested in research—inside or outside of academia—many expressed an interest in leading and guiding a research team rather than continuing to work at the bench. In response, nurturing leadership skills, understood broadly as leading people and projects, research governance and so on, became the main goal of LEAD. In practice, this is achieved through a combination of establishing peer networks amongst the participating postdocs, providing a framework for PIs to engage with their postdoc’s career development and dedicated workshops for cultivating leadership skills.

Here we discuss our experiences and provide insights for how postdocs, their PIs and institutes may successfully implement training programmes for cultivating the next generation of research leaders (Fig. 1). In particular, we want to challenge the narrative that a broader expertise is only catering for potential careers in industry and not worthwhile for an academic career. Cultivating skills such as people management, communication, teamwork, negotiation, stakeholder and diversity management and so on, are as valuable for leading research in academic organisations, as in industry. We also provide suggestions on how selection processes might be adapted to identify PI candidates with broader leadership qualities beyond a limited idea of research success. We believe that coordinated efforts to both nurture and select for leadership skills, can achieve a positive transition in postdoctoral training to ensure that postdocs have the necessary skills to excel as research leaders in academia or industry.

**Figure 1:**
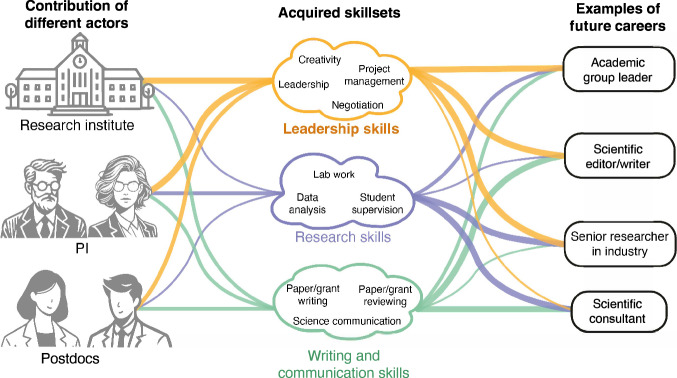
The influence of different actors (research institute, PI and postdoc peers) in shaping the development of a postdoc’s skill sets and how these different skill sets might influence their ability to work in different future career roles. The thickness of the lines represents the relative contribution from the preceding node.

“In particular we want to challenge the narrative that a broader expertise is only catering for potential careers in industry and not worthwhile for an academic career.”

## What can postdocs themselves do for their development?

Institutions and PIs clearly play an invaluable role in the training of postdocs, but postdocs themselves and as peer groups also have to become more active. Spontaneous support networks amongst postdocs are valuable, but these occur less frequently than during PhD programmes as more varied responsibilities both at work and home reduce the flexibility and availability for socialising. As such, more structured peer groupings can ensure a continuous support network and convince postdocs to carve out the time to discuss challenges and strategies in dealing with their new multifaceted role.

Such formalised peer groups have been a key element of LEAD from the onset of the fellowships, where postdocs recruited in the same call meet every month to support each other. Initially, the group members presented their science and then discussed topics selected by the group, such as publication authorship, student supervision and stress management. One potential refinement, without compromising the trust and open environment fostered within the peer group, is inviting a more senior scientist to the meetings or to parts thereof to lend their experience, particularly for addressing leadership challenges.

For postdocs, especially if fresh out of a PhD, the role can suddenly appear very expansive, with priorities shifting away from lab work towards supervising, teaching, grant writing, reviewing and organisational roles such as committee work just to name a few. For those seeking to excel in the role, knowing how best to allocate their finite time becomes an all-consuming problem. This challenge is often further increased by feelings of acute inadequacy when roles seen as essential are at times dropped as urgent grant deadlines or teaching commitments emerge. It is precisely for challenges such as these, that peer groups prove invaluable, as postdocs realise that they are not alone and can receive immediate and relevant advice from people who are or have recently been actively engaged in similar struggles.

“It is precisely for challenges such as these, that peer groups prove invaluable, as postdocs realise that they are not alone and can receive immediate and relevant advice from people who are or have recently been actively engaged in similar struggles.”

## What can PIs do to improve postdoctoral development?

Our immediate advice for PIs interested in improving career development for postdocs is to make sure that they consider a postdoc as a leader-in-training and that they encourage and support their postdocs’ independent research projects. This entails not only playing a mentor and, at times career coach, but also providing them with opportunities to take on diverse responsibilities other than research, such as supervision, grant/manuscript reviewing and committee roles. As part of LEAD, all host PIs have been encouraged to engage the LEAD fellows in such research-leadership tasks, facilitated by a career development plan. This helped to ensure their engagement varied for each fellow depending on the latter’s ultimate goals. When handled well, delegating responsibilities simultaneously trains postdocs whilst reducing administrative burden for PIs and provides better representation from more junior staff within organisations. Opening up the leadership role to postdocs should naturally come with providing them with more time and space to grow both personally and professionally. An inclusive leadership style and even co-management of research teams is already a growing tendency in some organisations and professions.

In addition to the managerial tasks discussed above, postdocs should also be included in the scientific ideation processes and the direction of research. In some places, ideas are still mainly seen as the purview of the PI; nonetheless, team efforts can strongly increase the chance of innovative ideas emerging from debate and discussions. As part of the LEAD programme, fellows receive training in creativity and collaboration to equip them with tools not only for their own work, but also to involve others in the creative process of research. Prior to the LEAD kick-off, all BRIC PIs were offered similar training with a focus on team creativity, but changing approaches take time and practice.

## What can institutes do to support postdoctoral development?

Depending on available resources, both financial and administrative, host institutes can play key roles in shaping opportunities for postdocs. At BRIC, support for the LEAD programme has been obtained from the H2020 Marie S. Curie COFUND programme. The co-funding covers only 40% of the salaries, but this has been sufficient incentive to kickstart the structured postdoc programme. LEAD is based on experiences within BRIC’s Postdoc Career Program (PCP; https://www.bric.ku.dk/phd-and-postdoc-programme/postdoc-career-programme/), where BRIC has offered transferable skills workshops and career seminars for up to 80 postdocs each year since 2015.

For institutions developing their postdoc training programmes, the simplest starting point is to organise meetings between arriving postdocs, either as larger peer groups—we recommend 6–8—or pairing them with a more senior postdoc ‘buddy’. Some care should be given to ensure that postdocs come from different research groups, as this not only benefits the institute by reinforcing cross-group interaction but can also allow postdocs to freely discuss challenges within their groups. Where feasible, appointing a dedicated administrator can help maintain momentum and ensure robust support with supplementary resources.

Prioritising training of postdocs in transferable skills is another key element of ensuring a good transition to a research-leader position, as well as broader career opportunities. Workshops around project management, creativity, self-leadership and innovation for creating startups, provided LEAD fellows with valuable skills and proved especially useful through guided processes for dedicated introspection around our individual leadership styles. We found different workshops to be variably valuable, depending on the respective deficits and interests of each attendee. As such, where possible we would advocate for workshop programmes to allow flexibility of course selection, as in BRIC’s PCP programme.

More broadly, we encourage institutions to go out of their way to create and advertise the contact points for postdocs to receive dedicated support, raise issues they face and use this information to steer organisation responses. Precisely because of the heterogeneous backgrounds and management/mentoring styles of different PIs, institutes should also look to formalise essential career development interventions for PIs to engage with. Ideally these guidelines would be drafted with consultation from both PIs and resident postdocs.

## How could institutes select for the next generation of leaders?

Finally, we reflect on how an organisation can select for aspiring research leaders by not only assessing publication records but also with a clear focus on leadership competencies. Currently, success in academia is often measured by largely one metric: high-impact publications. However, this alone does not guarantee the development of a successful leader of research. Core values such as empathy, respect for autonomy, and skills such as listening, resource management (human, financial, time), decision making, and creativity among others, are also part of the research leadership “package”. As such, institutes should particularly value when aspiring or established leaders have engaged in programmes geared towards the acquisition of management and leadership skills, as this highlights their efforts in expanding and understanding leadership beyond just research or personal success. Also, the motivation and planned approaches for leadership should be discussed during interviews, as well as how candidates plan to contribute to an engaging work environment.

In modern academia, there is increasing exposure for individuals to engage across diverse cultures, as research becomes more internationalised and inclusive. This does however come with challenges for effective communication, conflict resolution and broader management styles, especially when expected norms diverge. We recommend that institutions specifically prize evidence of interaction with multiple cultures whether demonstrated through career mobility, international collaborations or mentoring across diverse backgrounds.

At BRIC, group leaders are solely recruited through open calls and, in recent years, the recruitment process has included evaluation of a broad competence area using predefined scoring of the candidates. Competencies evaluated include not only research performance, future research plans and ability to attract grants, but also organisational contributions, leadership and management qualifications, including pedagogical competencies, supervision skills and building internal collaborations. These categories are individually evaluated, and candidates should address these in their application. Candidates may or may not have previous documentable leadership experiences, however, all are asked to reflect on their ability and ambition to develop their own academic leadership skills, to inspire, manage, and develop staff and to develop and secure a strong, open and inclusive research group. They should also address their ability and ambitions to collaborate and build internal relationships at the centre, and where they see themselves taking responsibility for specific as well as common institutional tasks. The use of clear criteria and required scoring in this variety of areas support fair and merit-based selection of candidates with a broad skill set in addition to a strong research profile. This approach has been refined now over three rounds of PI recruitment at BRIC—not directly linked to the LEAD programme, but strongly inspired from how the LEAD evaluation and selection process was organised—with a focus on unbiased, transparent and merit-based assessment.

The experiences from LEAD are still early days, but the programme already builds on several years of experience with talent development for early-career researchers at BRIC through the PCP. Based on these collated experiences, we strongly advocate the development and initiation of similar programmes to support postdocs in developing broader leadership competencies in research organisations. We see, for example, easy wins for institutions who are hiring five or more postdocs every year to support the establishment of peer-groups with basic recommendations around meeting every month and choosing topics of shared interests. Ideally, where funding permits, we find clear value in dedicated leadership skills workshops, with the caveat that different postdocs will have different concerns and needs. We also suggest mutually beneficial approaches for PIs to cultivate leadership skills within their mentees by steadily delegating portions of their own responsibilities as well as opening up the continual process of defining the research vision of the group.

However, ultimately the development of an independent researcher is of course driven by the individual. Therefore, postdocs should actively seek out support and resources to help them become and flourish as research leaders. As such, perhaps one of the easiest and most sensible ways for institutions to support postdocs is by providing clear signposts to resources, whether these focus on grant support, challenges they face or online resources for their career development. By nurturing leadership skills and effectively selecting for them when hiring in new positions, institutions can reinforce their reputations as exemplary places for postdocs to grow, ensuring talented trainees and group leaders are able to work together to more effectively push the frontiers of science. We believe that such measures should contribute to modernising research leadership and strengthening the work culture, well-being and engagement in academic organisations whilst driving scientific breakthroughs by including a larger, more diverse group of staff in ideation.

…“perhaps one of the easiest and most sensible ways for institutions to support postdocs is by providing clear signposts to resources, whether these focus on grant support, challenges they face or online resources for their career development.”

## Supplementary information

Peer Review File

