# Supplementary material for: Cultivating the next generation of leaders: How postdocs, principal investigators and institutes can nurture and select for leadership competencies
Source: EMBO Rep. 2024 Nov 12;25(12):5232–5. doi: 10.1038/s44319-024-00308-1 (PMC11624197; doi:10.1038/s44319-024-00308-1)
Supplement: Supplementary file 1 — Peer Review File [file 44319_2024_308_MOESM1_ESM.pdf]

EMBO reports

Cultivating the next generation of leaders

James William Bryson, Ülkü Uzun, Victor Oria, Jamie Auxillos, Iman Safari, Alexia Lopresti, Agnieszka Krzyzanowska, and Katrine Sonne-Hansen

Corresponding author(s): James William Bryson (james.bryson@bric.ku.dk) , Katrine Sonne-Hansen (katrine.sonne@bric.ku.dk)

Review Timeline:

Submission Date:	12th Oct 24
Accepted:	24th Oct 24

Editor: Holger Breithaupt

Transaction Report: This article was editorially reviewed.
